# On Albumen in the Treatment of Pulmonary Consumption

**Published:** 1877-07

**Authors:** E. L. Shurly

**Affiliations:** Detroit, Mich.


ART. II.—*On Albumen in the Treatment of Pulmonary Consumption.* By E. L. SHURLY, M. D., Detroit, Mich.

CONCLUDED.

1875, FEB. 6TH. CASE III.

Mrs. A. W. P.—æt. 25. Married. Tall and spare. Good hygienic surroundings. Has lost her father and three aunts on father's side with Phthisis Pulmonalis. Was tolerably well previous to marriage about a year ago, excepting a predisposition to "taking cold." Has had two attacks of slight hemoptysis, nine and seven months respectively; and since then, cough with more or less expectoration. Has some indigestion; slight nocturnal fever, preceded often about 5. P. M., by a chill, or chilly sensations; dysmenorrhoea which is increasing in severity; and dyspnoea upon exercising. Laryngoscopic examination shows slight reddening of the vocal cords, and granular pharynx.

Physical signs: Expansion of the right side of thorax only moderate. Percussion of right upper front shows resonance, but higher pitch. Respiratory percussion shows disparity between inspiratory and expiratory resonance on right side. Auscultation shows in right upper front a broncho-vesicular murmur and over back of right side from 3d rib down bronchial respiration with duller percussion sound. Auscultation shows also a prolonged expiration over this side especially in scapular and supra-regions. No abnormal transmission of heart sounds.

Treatment ℞. Glycerole of albumen ℥ XII. Syr. of hypophosphites co. ℥ iij. m. and take tablespoonful three times daily, also a warm spray of a solution of carbolic acid (gr. iij. ad. ℥.) to be inhaled three times daily, and a cathartic pill containing one half grain of Ext. belladon. to be taken just previous to the first day of menstruation, in order to mitigate the pain thereof.

Feb. 19th. It is noted that she now suffers from pain in the left side and hoarseness in the morning, but that the physical signs remain about as they were, and that she is also suffering from an acute nasal catarrh. The albumen was continued in the dry form, mixed with hypophosphite of soda.

Feb. 29th. It is noted that the cough and nocturnal fever have greatly abated, as also the pain during menstruation, and that she is improving. From this on she has been slowly improving, gaining in flesh and strength, while the physical signs have shown a slight clearing of the right lung, as evidenced by the more vesicular character of the respiration. She is now (Dec. 1876,) doing well, being able to attend to all of her household and other duties.

### Feb. 14th, 1875. Case IV.

Mary Kremer, æt. 17. Unmarried. Occupied by doing housework, pale, medium height and form stooped. Good hygienic surroundings and food. Good habits. Father a subject of bronchial asthma, but other members of family in good health. Was never very robust. Has had a cough and some expectoration more or ess for two years, with tendency latterly to night sweats. Voice nasal. Respiration frequent. Fever at night. Little appetite. Bowels constipated. Pain more or less in chest and epigastrium. Tonsils enlarged. Pharynx follicular and dry.

Physical signs: Auscultation shows mucous, and sibilant rales, and crackling over left infraclavicular and mammary regions, with bronchial respiration and prolonged expiration. On opposite side, sibilant rale upon forced inspiration, and rude respiration all over.

Treatment: ℞. Teaspoonful of dry albumen with five grains of Sodium hypophosphite four times daily; as many eggs to be consumed as possible, and one glass of ale at meals.

Dec. 1876. This treatment has been pursued closely ever since and she has slowly improved, all the symptoms of disease having greatly diminished. An examination of the lungs a few days ago shows Bronchial respiration and sibilant rales over upper left side of thorax, with considerable contraction. She still coughs and expectorates some, however, especially in the morning.

### 1875, July 15th. Case V.

Mrs. M. C. W.—æt. 20. Mother of one child. child; medium stature; pale and spare; lives on the bank of the river; surrounded by every comfort necessary to good living; although the house is very damp and stands near a distillery. Father healthy as also brothers and sister, but mother now lying ill of Phthisis Pulmonalis (advanced). Was in tolerable good health until a little over a year ago, when she contracted ague which continued to trouble her more or less until a month or so ago, but, apparently left her with a hacking cough, chronic nasal catarrh, and debility. She now has "spells of fever," and night sweats; little appetite; constipation; foetid breath; indigestion; cold feet; palpitation of heart; and nervousness. The cough is very troublesome during the night and the expectoration very little. Menstruation irregular but more painful. Is emaciating, and soon tires upon moderate exertion.

Physical signs: Nasal passages and Pharynx dry and covered more or less with crusts. Percussion sound little duller over left Infraclavicular and mammary regions. Respiratory percussion shows no disparity of sound between inspiration and expiration. Ausculation shows very feeble respiratory murmur and expiration equal in length to inspiration over these regions, with higher pitch, as compared with other side.

Treatment: Sol. of potass, pr. mang. (gr. ss. ad. ℥.) to be used with nasal douch every morning: five drops of Fowler's Sol. with three grains of quinine three times daily; three fresh eggs daily with wine, besides a diet of meat, milk, etc., as much as can be taken and digested and removal as soon as possible to another location.

Aug. 18th. It is noted that she has had since the first of the month more dyspnea and considerable pain in left side of thorax with increase of the hacking cough and that the physical signs remain about the same. The nasal catarrh worse. She has been unable to take more than two eggs daily from inability of the stomach to digest them. ℞, Counter irritation by means of repeated small blisters over side and front of thorax and to take one teaspoonful of Powd. albumen four times daily with two eggs in wine if possible. Continuing the use of douche.

Aug. 26th. She still has pain in chest, but less constant and severe, also fever during latter part of the day, and absolute loss of appetite. Stomach more quiet. Is fully under the effect of arsenic. She has not removed, and states that "mould" will form on boots and shoes in a short teme, when they are placed in any of the rooms having no fire.

Oct. 7th. Was better generally until a couple of days ago; coughs and expectorates considerable; fever and sweating every afternoon. Physical examination of chest shows moist crackling and subcrepitant rale over both upper lungs, but more in left side. Voice quite weak, and bronchophany to some extent in left infra-clavicular region. Treatment: To take five drs. daily of powd. albumen and two eggs daily if possible; three ozs. wine daily and two doses of quinine, three grs. each, for a few days.

From this on she slowly improved, and after removal to a new house in better locality improved more rapidly. During this time up to Feb. 1876, she has taken albumen either dry or in the natural condition in eggs. Dec. 9th, 1875, she had an attack of acute Bronchitis lasting about two weeks, but has continued to improve since, until now, Dec. 1876' she looks well; is fleshy; and quite vigorous—although the left lung is somewhat consolidated yet.

OCT. 21ST, 1875. CASE VI.

D. P. French Canadian, æt. 29. Shoemaker. Married. Tall and spare. Good family history. Works in a crowded, ill-ventilated shop, and lives in a crowded family. Has been unhealthy and weak for past three years, having had more or less dyspepsia and cough, the latter of which has been gradually growing worse. Had a severe hæmoptysis this afternoon following a severe paroxysm of cough.

Physical signs: Inspection shows sinking of supraclavicular region of right side; chest walls not very mobile, and respiratory acts frequent. Percussion shows upper right front to be a little duller; no difference shown by respiratory percussion. Auscultation shows feeble vesicular murmur in upper part of both lungs, but more bronchial in character on right side; also large mucous rales in both upper mammary regions and abnormal transmission of heart's sounds.

Treatment: ℞. Glycerole of albumen, tablespoonful three times daily, and Fl. ext. Ergotæ in thirty drop doses until hemorrhage ceases. To get out into the open air more, and sleep in an apartment constantly supplied with fresh air.

Dec. 1st, 1876. There was no more hemorrhage. The albumen treatment was continued more or less up to date, and he is now working at his trade again but not so manp hours daily. The upper right lung shows consolidation, he is not much fleshier but feels well and stronger than for four years past. His diet continues to be principally eggs and meat.

MAY 15TH, 1875. CASE VII.

J. G.—æt. 19. Printer; spare; tall; of Irish parents; comfortable home; temperate habits; very industrious. Good family history as far as known. Usually in tolerable health although never very strong. Was taken sick day before yesterday with chills and fever, and hacking cough. The chills, fever, cough, etc.

continued more or less for about ten days, during which time the cough continued and mucous and subcrepitant rales made their appearance, while the respiratory murmur grew more and more feeble, and the respiratory acts more frequent. He recovered very slowly from this attack, but continued coughing and expectorating, with more or less fever and sweating at night, and on June 25th, the Physical signs: showed a duller percussion note in Infraclavicular and Infrascapular region of right side, with Broncho vesicular respiration, and mucous and sibilant rales over both infraclavicular and mammary regions.

Treatment: consisted at first of quinine and laxatives, and subsequently of Emul. ol. marrh. et. album. About the middle of July he began doing light work again.

May 21st, 1876. It is noted that he gradually improved so that he began to feel quite well again until about two weeks ago, while returning from a visit to Wisconsin—crossing the lake—he contracted a severe cold and soon began coughing severely and expectorating considerable frothy blood, which has continued more or less up to this date (May 21st, 1876). It is noted that the physical signs on this occasion were—very feeble murmur, even upon forced inspiration, and abnormal transmission of heart's sounds, throughout the right upper front of thorax and rude respiration throughout lower lobe of right lung, with some large mucus rales in interscapular region. Percussion duller over upper left side. Treatment consisted of tablespoonful of Glycerole of albumen with five grs. of sod. hypophosphite four times daily. Fl. ext. ergotæ, and good ale. The hemorrhage soon ceased and he continued to improve, but never gaining much in weight, until now (Dec. 1876,) he feels quite well and is able to do "a light day's work." The upper right lung, however, remains changed from the normal condition, and he still coughs a very little. He has been taking the albumen more or less up to last month (Nov. 1876), and was recommended to continue its use more or less.

### 1875, Nov 2d. Case VIII.

Wm. N.—æt. 40. Policeman, formerly a miner. Married. Tall and very thin. American. Dwelling not very clean or airy.

Temperate habits, though in the habit of drinking some whiskey, and by virtue of occupation is up nights more or less, when he often contracts "a cold." Family history not ascertained, was in good health and fleshy until about a year ago, when he contracted "a cold" which, as he says, has never left him. Has chills: fever, and sweating; is emaciating and gradually growing weaker; appetite poor; bowels constipated, and considerable cough and expectoration.

Physical signs: Percussion sound duller over infraclavicular and mammary region of right side and more resonant than in health over left side generally; mucus (large and small) rales and sibilant rales over both sides; bronchial respiration and bronchophony over upper right lung, and feeble murmur elsewhere.

Treatment: Albumen, muriate of ammon. and quinine.

This patient has been under treatment ever since, principally by albumen and sodium hypophosphite; and although he has from time to time suffered from acute attacks of bronchitis, he has steadily gained a little, and now is able to attend to his duties as policeman, with but little intermission. The right lung has contracted considerably as shown by the form of the thorax and the physical signs show bronchial respiration in upper right lung with some vesicular (probably) emphysema in left lung. He now takes fresh eggs and whiskey daily in addition to ordinary meals.

### 1875, Dec. 2d. Case IX.

Mrs. A. P.—Norwegian; mother of five children; æt. 32. Tall, "large framed" woman but spare. Resides on the bank of river, but the ground on which house stands is well drained. Has not had any ague for several years. In comfortable circumstances though of frugal habits. Family history, as far as known, good. Has been out of health for past five months, during past two months having cough with little expectoration, and during last two months having nocturnal fever and sweating, but not preceded by chills at all. Has loss of appetite; constipation usually; sense of great debility, considerable cough and sometimes bloody expectoration and is emaciating quite rapidly.

Physical signs: Percussion shows dullness over both scapular and infrascapular regions, also over infraclavicular and upper mammary regions of right side. Auscultation shows broncho-vesicular respiration in both upper fronts, and behind in both suprascapular and scapular regions vesicular quality nearly absent. In infrascapular regions the respiration shows nearly normal vesicular quality. Sibilant rale and bronchial voice in right upper front.

Treatment: Four tablespoonfuls daily of gly. albumen; meat diet, eggs and fresh air, together with the donning of flannel underclothing. This treatment was continued "right along" and now, Dec. 1876, she is stronger and weighs about fifteen pounds more than for three years past. She still coughs a little, especially in the morning. Physical signs show no rales, but still a rude respiration over upper right front, and a more vesicular quality over scapular and infrascapular regions of right side.

As the abstracts of cases already given occupy considerable space and as enough detail has been given to show the plan of observation and diagnostic points entertained, I shall therefore in the following cases only give the more salient points, and thus reduce their narration to the least possible amount of matter.

1875, DEC. 15TH. CASE X.

R. F.—æt. 19; tall and thin; clerk; dissipated periodically; good hygienic surroundings; lost two brothers of Phthisis Pulmonalis. Has had one attack of hemoptysis and "dry cough" for a year which is growing worse, also nocturnal fever.

Physical signs: Percussion note little duller in upper left thorax. Auscultation. Dry crackling, sibilant and subcrepitant rale over both upper lungs—more marked over left. Bronchial respiration and some mucus rales in upper left side; inspiration and expiration same length.

Comments. Was under treatment nine months principally by albumen and gradually improved until now he is at business and feels tolerably well. Weight not increased much.

1876, JULY 3D. CASE XI.

Mrs. L.—æt. 39; mother; suckling babe one year old at night; very slender and sallow. Lives in small apartments and is poor.

Had a cough for a long time when young. Brothers and sisters subject to cough. Was quite well until three months ago, when slight hemoptysis came on followed by a hacking cough which has continued since. Expectorates some in morning. Has slight nocturnal fever. Is emaciating.

Physical signs: Percussion about same over both sides. Auscultation. Very feeble vesicular murmur both sides, especially in right infrascapular and infra-axillary regions. Expiration prolonged and jerky. Abnormal transmission of heart's sounds.

Comments: Is still under treatment by albumen. "Is holding her own."

1876, JAN. 22D. CASE XII.

M. D. W.—æt. 26; tall, spare; gentleman brought up in affluence; dissipated; whole family unsound physically, being especially subjects of nervous disorders. Was in tolerable health until about four months ago when he "took cold." Is now under great mental pressure. Is very nervous, sleeps badly and sweats. Has dry cough almost constantly. Emaciating; little appetite; respiration and pulse very frequent.

Physical signs: Percussion note duller over right infraclavicular region. Auscultation. Broncho vesicular respiration over upper left front. Bronchial respiration over upper right front; prolonged expiration; crust crackling, sibilant, and small gurgling in upper right infraclavicular region.

Treatment: Teaspoonful of albumen with two grs. calc. hypophosphite four times daily, and three eggs daily. To go south as soon as possible.

Comments: He continued this treatment during his sojourn in the South and gradually improved so that this winter he is in Wisconsin, and, I hear, very much improved, though not well. He is still pursuing the same treatment.

1876, FEB. 9TH. CASE XIII.

Miss J. M.—æt. 20; comfortable surroundings; medium stature; quite thin. Very sickly mother but healthy father. Brothers and sisters all living. Has been subject to a cold on and off for more than a year. Has had constant hacking cough for about three months, and is emaciating. More or less pain in chest. Dysmenorrhœa and quantity scanty. Easily sweats, no fever though.

Physical signs: Respiratory murmur very weak in both lungs, prolonged expiration in upper part of left, with dry crackling. No difference shown on percussion.

Treatment: Emil. albumen et ol. morrh; out door exercise, and belladonna at menstrual period.

Comments: She feels quite well now and has no dysmenorrhœa. Has gained a little in flesh. Examination of chest on 3d of Nov. 1876, shows no adventitious sounds, and a clearer murmur, although it is yet weak. She has been taking on an average three eggs daily for last three months.

1876, MAY 29TH. CASE XIV.

Mrs. J.—æt. 32. Tall and spare. Does little work of any kind. Comfortably situated in life. Family history good, though brothers and sisters are not very well. Edges of lids reddened. Never very strong. Had a slight hemoptysis or two, a couple of weeks ago, which was preceded for about two months by hacking cough. Is very nervous. Menstruation scanty, insomnia; indigestion; emaciating; and growing weak; constipated and cough almost constant. Pain in left side.

Physical signs: Broncho vesicular murmur over both upper parts of lungs, higher pitch in left side, also bronchial respiration in patches over upper part of both lungs. No difference of percussion note. At a subsequent examination a sibilant rale was discovered in left infraclavicular and suprascapular region.

Treatment: More fresh air, albumen, and quinine.

Comments: She has been under the albumen treatment ever since with the addition of inhaling a warm spray impregnated with either, Fl. ex. of courium. or carbolic acid. She still coughs, especially in damp weather, but up to this time has gained ten pounds in weight and feels much stronger.

I have some other cases under treatment—principally by albumen—which, as they came under my care more recently will be omitted from this report.

This plan of treatment was partially tested in a case of acute

Tuberculosis, but with no positive results, and in three cases of advanced Phthisis, with probably no other effect than to diminish somewhat the nocturnal fever and sweating. But in a case of marasmus, so called, or "tuberculosis of the mesenteric and bronchial glands" of considerable duration, in which cod liver oil had been extensively used, the effect of albumen in quite large doses was very satisfactory. Also, in a case of severe gangrene of the lungs, it undoubtedly had the effect of prolonging life.

